# Disaster Effect Among Preadolescents Along With a Search for an Evidence-Based Preventive Approach: A Systematic Review

**DOI:** 10.7759/cureus.41497

**Published:** 2023-07-07

**Authors:** Nirupam N Sahu, Jaya Gawai

**Affiliations:** 1 Child Health Nursing, Smt. Radhikabai Meghe Memorial College of Nursing, Datta Meghe Institute of Higher Education & Research, Wardha, IND; 2 Mental Health Nursing, Smt. Radhikabai Meghe Memorial College of Nursing, Datta Meghe Institute of Higher Education & Research, Wardha, IND

**Keywords:** positive psychology, evidence based preventive approach, disaster, stress, preadolescent

## Abstract

The mental health of preadolescents is crucial for safeguarding our future. The purpose of this study was to assess evidence-based preventive measures for reducing disaster-related stress among preadolescents. The study design involved a systematic review of articles published before April 2023. For data sources, we conducted searches on PubMed, Google Scholar, Cochrane, the National Library of Medicine, and other relevant resources, following the Preferred Reporting Items for Systematic Reviews and Meta-Analysis (PRISMA) standard flow diagram for the systematic article review. Of 1,531,932 studies identified in the database search, 23 articles met the inclusion criteria, including one conducted in India. Disaster-related stress was found to be prevalent in preadolescents, with adverse effects that are particularly pronounced in this age group compared to other children. The stress experienced during the pandemic has had a negative impact on preadolescents' psychological well-being, emphasizing the need for focused care to protect them. Various preventive approaches have been identified to alleviate the suffering of preadolescents. Among the studies reviewed, a total of seven studies demonstrated the impact of disasters on the mental health of children, providing evidence that children have been significantly affected by the pandemic. Additionally, five studies highlighted preventive interventions to mitigate the impact of disasters on children's mental health, underscoring the necessity for psychological interventions. Several studies also revealed that preadolescents are more susceptible to disaster-related stress due to their developmental stage. Consequently, preventive measures were investigated to address this stress, specifically among preadolescents. In conclusion, continuous research on disaster-related stress is essential to determine the extent of stress experienced and to identify evidence-based measures, such as positive psychology, to mitigate its consequences. This not only protects the mental health of preadolescents but also safeguards our future generations from the burdens of distress.

## Introduction and background

Disasters, whether natural or man-made, have the potential to impact individuals of all ages significantly. Among the vulnerable population, preadolescents, who were in the transitional stage between childhood and adolescence, were particularly susceptible to the adverse effects of disasters on their mental health and overall well-being. Understanding the impact of disasters on preadolescents and identifying effective preventive approaches were paramount for safeguarding their future mental health [1,2]. This systematic review aimed to explore the disaster effect on preadolescents and sought evidence-based preventive approaches to mitigate the negative consequences. By synthesizing existing research in this field, we intended to contribute to understanding effective interventions and strategies that could effectively reduce disaster-related stress among preadolescents [2-4].

Preadolescence, typically 9 to 12 years, marked a critical developmental phase characterized by significant physical, cognitive, and emotional changes. During this stage, commonly referred to as the "tween" years, preadolescents navigated the complexities of transitioning from childhood to adolescence, grappling with identity formation, social interactions, and the onset of puberty. Such profound developmental changes made them particularly vulnerable to the disruptions caused by disasters [5-7].

The aftermath of disasters could have profound psychological and emotional impacts on preadolescents. They may have experienced fear, anxiety, grief, and a loss of security. The disruption of their daily routines, separation from loved ones, and exposure to traumatic events could have further exacerbated their distress. Consequently, there was a critical need to identify preventive measures that could effectively mitigate the adverse effects of disasters on preadolescents' mental health and well-being [8].

In this systematic review, we meticulously analyzed a range of studies, including observational, longitudinal, and cross-sectional studies, to gather comprehensive evidence on the impact of disasters on preadolescents. Additionally, we critically evaluated interventions and strategies implemented to prevent and alleviate disaster-related stress in this vulnerable population. The findings from this review provided valuable insights into the most effective approaches for reducing the impact of disasters on preadolescents' mental health. This knowledge could inform policymakers, educators, and mental health professionals in developing targeted interventions and support systems to protect preadolescents in the face of disasters.

## Review

Methodology

Data Sources

The Preferred Reporting Items for Systematic Reviews and Meta-Analysis (PRISMA) standard flow diagram showed a comprehensive search of multiple databases, including PubMed, Google Scholar, Cochrane, the National Library of Medicine, and other relevant resources [3]. The objective was to systematically review articles focusing on disaster stress among preadolescents and evidence-based preventive approaches to safeguard our future citizens. The search yielded 15,31,932 studies, from which 23 articles met the inclusion criteria, including one study conducted in India (Table 1).

**Table 1:** Detailed Search Strategy in Four Data Sources

Sr. No.	Combination of mesh words and keywords	Site	Search results
1	Disaster stress among children	Google Scholar	15,30,000
2	Disaster stress among children and preventive approach	PubMed	41
3	Disaster stress among children	PubMed	1805
4	Disaster stress among children	Medline	32
5	Disaster stress among children and preventive approach	Medline	3
6	Disaster stress among children	Cochrane library	0
7	Disaster stress among children	Cochrane library	49
8	Disaster stress among children and preventive approach	Cochrane library	2

Study Selection (Inclusion and Exclusion Criteria)

Following the initial search, the removal of duplicate studies was performed. The remaining studies underwent a screening process by the researchers based on titles and keywords. Specifically, studies that examined the impact of disasters on preadolescent children and those that focused on preventive approaches to mitigate the psychological effects of disasters were included. Studies centered solely on adults and preventive approaches unrelated to mental health were excluded. Articles with limited availability of free full-text versions overlap with other studies, research conducted on animals, and incomplete data were also excluded.

Analysis of Study Quality

Using standardized tools to evaluate their quality, we thoroughly assessed 12 carefully chosen studies. These studies were categorized as low, moderate, or high quality, and only those of sufficient quality were included in the review. The Newcastle-Ottawa Scale for observational studies was used to assess the quality of these 12 studies (Table 2).

**Table 2:** Summary of the Newcastle-Ottawa Risk-of-Bias Tool for Observational Studies The Newcastle-Ottawa Scale for observational studies [16].

Sr. No.	Study Reference	Selection of Study Groups	Comparability of Groups	Ascertainment of Exposure	Outcome Assessment	Total Score	Quality
1	O’Sullivan et al., 2020 [4]	2	2	2	3	9	High
2	Villanueva et al., 2020 [5]	2	2	2	2	8	High
3	Furr et al., 2010 [6]	1	2	2	3	8	High
4	Saurabh K, Ranjan S, 2020 [7]	2	1	2	3	8	High
5	Adams et al., 2021 [8]	2	2	1	3	8	High
6	Spinelli et al., 2020 [9]	1	1	2	3	7	Moderate
7	Gilsbach et al., 2021 [10]	2	2	2	1	7	Moderate
8	Kubo T, Masuyama A, 2022 [11]	1	2	2	3	8	High
9	Jones et al., 2021 [12]	2	1	2	3	8	High
10	Amin et al., 2020 [13]	1	1	2	3	7	Moderate
11	Pfefferbaum et al., 2019 [14]	2	2	1	3	8	High
12	Pfefferbaum et al., 2014 [15]	1	1	1	3	6	Low

**Figure 1:**
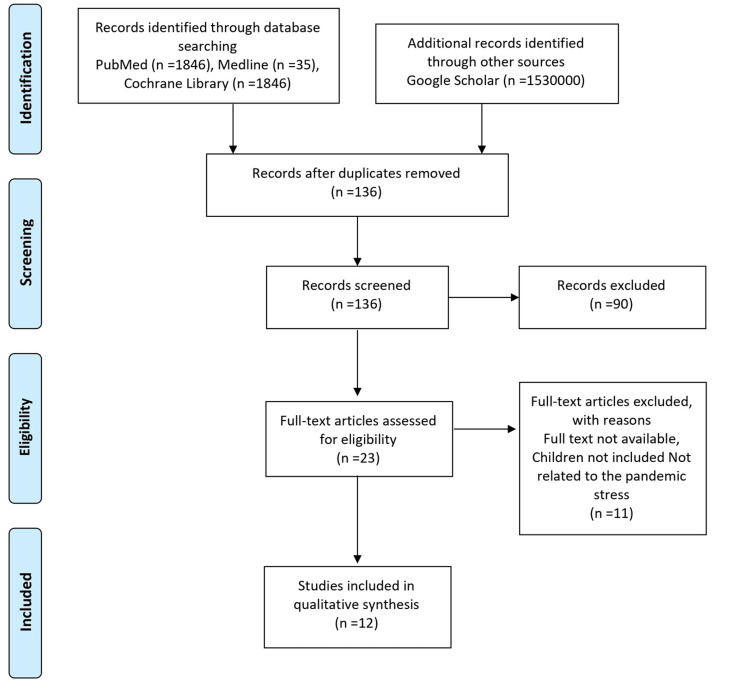
Flowchart of Literature Review Search per Preferred Reporting Items for Systematic Reviews and Meta-Analysis (PRISMA 2020) Guidelines Source [3]

A total of 136 studies relevant to this study were initially identified. Out of these, 90 studies were retrieved from PubMed, while an additional 46 studies were sourced from Google Scholar using the keywords "disaster stress among children" and "preventive approach." After a comprehensive examination, 23 articles were deemed pertinent to the current study. Ultimately, a final selection of 12 studies was made to be included in the review. As shown in Figure 1, the PRISMA flowchart illustrates the literature search strategy and the subsequent selection process.

Among the chosen studies, seven investigations involving approximately 76,181 participants focused on understanding the impact of disasters on children's mental health. These studies provide compelling evidence that children are significantly affected by the ongoing pandemic, as highlighted in Table 3.

**Table 3:** Studies Showing the Effect of the Disaster on the Mental Health of Children PTS: Post-traumatic stress

Sr. No.	Author and Years	Country	Study Design	Target Population	Total Participant (N)	Exposure	Intervention	Outcome
1	O’Sullivan et al., 2020 [4]	Ireland	Qualitative Study-Interpretative Phenomenological Analysis	Child and Adolescent	94	COVID-19 Pandemic	-	Adverse mental health effects, including feelings of social isolation, depression, anxiety, and increases in maladaptive behavior
2	Villanueva et al., 2020 [5]	Spain	Longitudinal prospective design	Preadolescent and adolescent	381	No exposure	TMMS-24 PSS-4 Perceived Stress Scale Rosenberg Self-Esteem Scale (RSE; Satisfaction with Life Scale (SWLS)	Trait emotional intelligence, self-esteem, and low perceived stress were related in the expected direction to life satisfaction and somatic complaints. Findings support a specific pathway to improve well-being in preadolescents.
3	Furr et al., 2010 [6]	Washington	Meta-analysis	Youth	74,154	Disaster	-	Youths are vulnerable to appreciable PTS after the disaster, with pre-existing child characteristics and aspects of the disaster experience.
4	Saurabh K, Ranjan S, 2020 [7]	India	Survey	Children 9-18 Years	121	COVID -19	-	Compliance and mental health problems can be improved by providing adequate financial support and enhanced knowledge about pandemic planning.
5	Adams et al., 2021 [8]	United States	Online survey	Parents of age5-18 years	433	COVID -19	-	Parent stress increased substantially during COVID-19 and has not returned to pre-COVID-19 levels, suggesting enhanced mental health resources and support are needed.
6	Spinelli et al., 2020 [9]	Italy	Online survey	Parents of age2-14 years	854	COVID -19	-	Policies should consider the implications of the lockdown for families’ mental health, and supportive interventions for the immediate and future should be promoted.
7	Gilsbach et al., 2021 [10]	Germany	Comparative study	Children (6-18 Years)	144	COVID -19	-	Children and adolescents with a mental illness, particularly female children and individuals with a depressive disorder, are at an increased risk of suffering from pandemic-associated psychological distress. Adequate mental health care options, such as telepsychiatry, are indispensable

Additionally, five studies, with a collective participant count of approximately 47,691, centered on preventive approaches to mitigate the adverse effects of disasters on children's mental health. These findings underscore the necessity of implementing psychological interventions to reduce the pandemic's impact on children, as presented in Table 4.

**Table 4:** Preventive Approach for the Disaster Effect and Its Outcome PTSD: Post-traumatic stress disorder; PTS: post-traumatic stress

Sr. No.	Author and Years	Country	Study Design	Target Population	Total Population (N)	Exposure	Preventive Approach	Outcome
1	Kubo et al., 2022 [11]	Japan	Intervention study	Preadolescent	248	COVID-19	Self-monitoring and psycho-education	School-based interventions that intend to raise children’s awareness of COVID-19 promote their healthy development and adaptation to crises within the school.
2	Jones et al., 2021 [12]	United States	Systematic Review	Adolescent	40076	COVID-19	-	Social support, positive coping skills, home quarantining, and parent–child discussions seem to impact adolescent mental health during this crisis period positively.
3	Amin et al., 2020 [13]	Pakistan	Pilot study	Children	75	Flood-Impacted Children	SSET program. Lesson plans, worksheets, and materials.	Support for Students Exposed to Trauma (SSET) programs is found effective in reducing PTSD symptoms and building resilience and social support among children living in flood-affected rural areas of Southern Punjab, Pakistan.
4	Pfefferbaum et al., 2019 [14]	United Kingdom	A Review and Meta-Analysis	Youth	4662	Mass trauma	-	Interventions can alleviate PTS and enhance functioning in children exposed to mass trauma.
5	Pfefferbaum et al., 2014 [15]	United States	A Review and Meta-Analysis	Children and Adolescent	2630	Disaster and terrorism	-	Children and adolescents receiving psychological intervention fared significantly better than those in control or waitlist groups concerning PTSD symptoms

Discussion

The present systematic review aimed to investigate the impact of disasters on preadolescents and explore the use of positive psychology as a preventive approach. Our review identified only 12 studies that met the inclusion criteria in examining the effects of disasters on preadolescents and the effectiveness of positive psychology interventions. These studies highlighted the need for more research to establish evidence-based preventive approaches for reducing disaster-related stress among preadolescents.

One study by Gillies et al. focused on emotional treatment for children and teenagers who experienced a calamity [17]. The findings revealed that these young individuals were at a higher risk of developing tension, trauma, aggressive behavior, and psychological distress, all interconnected with their self-perception and overall well-being. The study analyzed short-term, medium-term, and long-term data from 51 experiments involving 6,201 participants. Various disasters were investigated, including physical injuries, environmental calamities, and societal violence. The study highlighted the effectiveness of psychological therapies, particularly cognitive-behavioral therapy (CBT), in reducing post-traumatic stress disorder (PTSD) symptoms and improving psychological well-being among preadolescents and teenagers [16].

Another study by LeMoult et al. explored the relationship between stress experienced before maturity and the onset of extreme depression in youth [18]. The study investigated eight specific types of stressors, such as physical harassment, environmental calamities, and emotional abuse, and their association with the development of major depressive disorder (MDD) before adolescence. The findings demonstrated that exposure to stress before maturity significantly increased the risk of MDD in youth. This study provides crucial evidence that the negative impact of stress before maturity on the likelihood of developing extreme sadness occurs early in life, emphasizing the importance of early intervention and support for at-risk children and teenagers [18].

Gillies et al. conducted another study focusing on psychological therapies for treating PTSD in young individuals who have experienced traumatic events [19]. The study compared various psychological therapies to control groups or pharmacological treatments. CBT emerged as the most effective psychological intervention, leading to significant improvements in PTSD symptoms in the short term and long term. However, the study acknowledged the limitations of the available research, such as methodological biases and a limited number of studies. Further research is needed to explore different types of injuries and their specific impacts on PTSD diagnosis [19].

Johannesson et al. investigated the long-term effects of calamity-related loss on individuals who experienced high sea waves caused by an earthquake [20]. The study focused on the emotional distress and psychological well-being of individuals who lost close relatives due to the disaster. The findings revealed that individuals who faced the calamity experienced lifelong sadness and increased psychological distress, particularly those who lost young relatives. The study emphasized the need for continued assessment and long-term support to overcome the psychological effects of post-disaster loss [20].

Hassan et al. conducted a study on children's reactions to a flood disaster in Kashmir [21]. The study assessed the post-traumatic stress (PTS) symptoms of child survivors using standardized questionnaires. The findings indicated moderate to high-stress levels among the children, highlighting the need for psychological interventions to address their emotional well-being.

Parvathy explored resilience and coping among children affected by a severe flood in Kerala [22]. The study examined the relationship between resilience and coping strategies in children. The findings revealed a significant correlation between resilience and coping, suggesting that promoting resilience can positively impact children's ability to cope with disaster-related trauma. The study emphasizes the importance of implementing intervention programs that enhance resilience and coping skills among affected children [22].

Amin et al. evaluated a disaster relief program to reduce PTS symptoms and promote flexibility and social support among young individuals in rural areas affected by cyclones [13]. The program included various components, such as community engagement, psychoeducation, and skill-building workshops. The findings demonstrated the program's effectiveness in reducing PTS symptoms and improving social support and flexibility among the participants [1].

A qualitative study by Somasundaram et al. in Northern Sri Lanka highlighted the psychological and emotional issues experienced at the individual, household, and societal levels in a post-war context [23]. These issues encompass grief, insecurity, self-harm, suicidal tendencies, economic hardships, adolescent and unwanted pregnancies, alcoholism, child maltreatment, gender-based violence, and vulnerability. The study also identified protective factors such as family support, female leadership, cultural beliefs, and traditions that contributed to resilience. To address these challenges and promote flexibility, the authors suggested community-based programs that raise awareness, provide support, and educate individuals on managing psychological and emotional issues [23].

The study by Tam et al. focused on the long-term prospects and protective factors associated with traumatic stress among forcibly deprived children [24]. The findings indicated a high prevalence of PTS among these children, with rates ranging from 20% to 48.7% at baseline and varying over time. The study identified risk factors including exposure to disaster or other stressors, older age, and previous psychopathology. The authors emphasized the importance of routine screening for mental health in this vulnerable group and early intervention to prevent the recurrence and exacerbation of psychological issues [24].

A systematic review by Rubens et al. explored the impact of environmental disasters on adolescent internalizing and externalizing issues [25]. The meta-analysis of various studies revealed a statistically significant relationship between exposure to natural calamities and the presence of suppressing and expressing attitude issues among teens. The study also highlighted the influence of socioeconomic factors, with a stronger association observed in countries with lower human development index positions. The findings underscored the need to comprehensively understand post-disaster mental health outcomes to inform holistic intervention strategies [25]. These studies shed light on the impact of disasters on preadolescents and highlight the significance of psychological interventions and positive psychology approaches in mitigating the negative consequences of such events. However, further research is needed to establish more robust evidence and develop tailored preventive strategies to promote the psychological well-being of preadolescents in the face of disasters.

## Conclusions

In conclusion, it is evident that children, particularly teenagers, represent the most vulnerable group due to the physiological and psychological changes that occur during this critical developmental period. Disasters have been shown to have an amplified impact on this age group, further emphasizing the need for increased attention and early intervention to safeguard the well-being of our future generation. Among the various preventive measures examined in this review, positive psychology has emerged as a successful approach in significantly reducing disaster-related stress. Positive psychology offers a promising avenue for mitigating the adverse effects of disasters on teenagers by focusing on enhancing happiness and emotional well-being and developing strengths and virtues.

However, it is crucial to acknowledge that further studies are needed to establish an evidence-based preventive approach that can effectively reduce the prevalence of disaster stress and protect the psychological health of our future generation. By conducting more research in this area, we can gain deeper insights and develop targeted interventions that effectively address teenagers' specific needs and vulnerabilities during and after disasters. Safeguarding teenagers' mental health and well-being in the face of disasters is of utmost importance. By implementing evidence-based preventive approaches, we can empower this vulnerable group to navigate the challenges and disruptions brought about by disasters, promoting their resilience and ensuring a healthier future for future generations.
